# Privacy-preserving logistic regression with secret sharing

**DOI:** 10.1186/s12911-022-01811-y

**Published:** 2022-04-02

**Authors:** Ali Reza Ghavamipour, Fatih Turkmen, Xiaoqian Jiang

**Affiliations:** 1 University of Groningen, Nijenborgh 9, Groningen, Netherlands; 2 UTHealth School of Biomedical Informatics, The University of Texas, Houston, USA

**Keywords:** Logistic regression, Secret sharing, Multi-party computation, Privacy-preserving, Newton–Raphson

## Abstract

**Background:**

Logistic regression (LR) is a widely used classification method for modeling binary outcomes in many medical data classification tasks. Researchers that collect and combine datasets from various data custodians and jurisdictions can greatly benefit from the increased statistical power to support their analysis goals. However, combining data from different sources creates serious privacy concerns that need to be addressed.

**Methods:**

In this paper, we propose two privacy-preserving protocols for performing logistic regression with the Newton–Raphson method in the estimation of parameters. Our proposals are based on secure Multi-Party Computation (MPC) and tailored to the honest majority and dishonest majority security settings.

**Results:**

The proposed protocols are evaluated against both synthetic and real-world datasets in terms of efficiency and accuracy, and a comparison is made with the ordinary logistic regression. The experimental results demonstrate that the proposed protocols are highly efficient and accurate.

**Conclusions:**

Our work introduces two iterative algorithms to enable the distributed training of a logistic regression model in a privacy-preserving manner. The implementation results show that our algorithms can handle large datasets from multiple sources.

## Background

Patient data (i.e., medical records and genomes) are being collected at a rapid pace around the world, and the volume of data is exponentially growing. In order to boost the power of statistical analysis and the robustness of machine learning models over these data sets, more samples are needed. One way the hospitals and research institutions can tackle the scarcity of data is to collaborate with each other by sharing data and findings in a central location. The key advantages of data sharing and/or collaborative data processing include more accurate disease identification and diagnosis, risk calculation for a certain disease, and therapeutic discovery.

Various data analytic techniques can be employed to infer information from a given datasets. The logistic regression model [1], one of the most popular prediction models, is now widely used in medical research. Logistic regression estimates a particular event’s probability based on previously observed data. More specifically, the value of a binary variable is predicted based on several independent variables. For example, a logistic regression can be trained for the identification of a malignant breast cancers based on tumor size, patient age, blood type, and genetic inheritance [2]. Statistical models need a sufficiently large sample size to achieve a desired level of performance in data analysis and to make more accurate predictions [3]. It is thus beneficial to combine and compare data from different sources to ensure generalisability in data representation. However, collecting data from multiple sources often raises concerns about privacy. Due to institutional policies and legal restrictions, hospitals and medical centers are often reluctant to share  their sensitive data (i.e., especially patient-level information) with other institutions. Therefore, it is essential to employ privacy-preserving solutions when making statistical inferences over shared data [4].

In this paper, our particular focus is on enabling logistic regression between multiple data holders  in a privacy-preserving manner. We assume that the data are horizontally partitioned, which indicates that the data holders have precisely the same variables but different values for those variables. Moreover, we assume that Newton-Raphson method is used to estimate model parameters. Based on these assumptions, we propose two methods to train a logistic regression model under different security assumptions. Thus, the main contributions of this paper include:

- A novel privacy-preserving algorithm for computing logistic regression models that is highly accurate and has an acceptable efficiency.
- A second algorithm that is highly efficient but less accurate due to the use of multiple approximations.
- Implementation of the proposed algorithms in both honest and dishonest majority security settings.
- Evaluation of the proposed protocols on various real-world and generated synthetic datasets.

## Related work

The literature contains a several research works that present privacy-preserving methods for the computation of logistic regression across multiple data holders. In general, the common approach has been the implementation of various steps in logistic regression by using primitives from cryptographic techniques such as multi-party computation, homomorphic encryption, and differential privacy. However, due to the complexity of the underlying secure computation primitives and the way they are employed, the existing methods suffer from multiple drawbacks regarding scalability and accuracy.

The Grid Binary LOgistic REgression (GLORE) model was developed by Wu et al. [5] to support privacy-preserving logistic regression in a distributed setting. GLORE estimates global model parameters for horizontally partitioned data without necessarily sharing patients. Instead of directly sharing sensitive data with other institutes, the decomposable intermediary results with significantly less sensitive information are transferred to build a global training protocol for logistic regression. However, in their proposed methods, sensitive data could be leaked due to disclosure of the information matrix and summary statistics.

Shi et al. [6] proposed a secure multi-party computation framework for grid logistic regression (SMAC-GLORE), which protects the patient data’s confidentiality and privacy. SMAC-GLORE preserves the intermediate results with the help of garbled circuits during iterative model learning. Various approaches, such as secure matrix multiplication and addition, and fixed-Hessian methods, have been employed to estimate the maximum likelihood function. Nevertheless, due to the garbled circuit constraints, SMAC-GLORE cannot handle a large number of records and features. Furthermore, it uses the Taylor series approximation approach to evaluate the sigmoid function, which causes accuracy loss in the final result.

Xie et al. [7] developed PrivLogit, which performs distributed privacy-preserving logistic regression and uses Yao’s garbled circuits and Paillier encryption. PrivLogit needs the data owners to perform computations on their data before encryption to compute parts of a logistic regression matrix resulting with an expensive computational cost to calculate the intermediate results.

SecureML [8] was one of the fastest protocols for privacy-preserving logistic regression models training based on secure MPC. The SecureML protocol is divided into an offline (to generate and distributing multiplication triples) and an online phase. SecureML employs a multiplication protocol based on a straightforward and efficient security setting introduced by Beaver [9]. To compute the activation functions, they also introduced a new comparison-based activation function that converges to 0 and 1. Unlike our work that employs the Newton Raphson optimization method, SecureML focuses on the mini-batch gradient descent.﻿

Cock et al. [10] proposed an information-theoretically privacy-preserving model training protocol that employs secret sharing-based building blocks such as distributed multiplication, distributed comparison, bit-decomposition of shares. Similar to SecureML, their proposed protocol requires multiplication triples distributed during a setup phase with/or without a trusted authority. Unlike SecureML, which is secure in the computational context, they engage in the information-theoretic model using secret sharing-based MPC and employ commodity-based cryptography [9] to decrease the number of communications.

In addition to MPC-based solutions, two popular methods have been considered. The first one is homomorphic encryption [11], which allows for computation to be performed over encrypted data.This method has been applied to privacy-preserving logistic regression in various works [12–18]. In most of these mentioned works, polynomial approximations need to be made to evaluate non-linear functions in machine-learning algorithms. The second method is differential privacy, a universally accepted mathematical structure for protecting data privacy. The main application of differential privacy in machine learning is when the model is publicly published after training in a way that personal data points cannot be distinguished from the released model [19–22].

## Preliminaries

In this section, we first present our system models along with the security assumptions. We then provide an overview of the secret sharing protocols used in our work and briefly present logistic regression.

### System model

Inline with the literature [8, 23], we consider a set of input parties that aim to train a logistic regression model on their sensitive data and assume that the data are horizontally partitioned among the input parties, i.e. each independent database contains only a sub-population. The input parties send secret shares of their inputs to a set of computation parties in a setup phase that is performed only once.

With regards to security, we consider three concerns when training a privacy-preserving logistic regression model. The first concern is the risk of colluding two or more parties involved in the protocols against other parties. The collusion might happen when two or more computation parties try to reconstruct the original data in the protocol by pooling their views. A computation party’s view comprises its private input, generated random data, intermediate values, and a list of all messages received during the execution of the protocol. For instance, a malicious computing party may try to modify the messages to break the privacy of the input parties. To do this, the computing party needs information from other parties to see how its changes affect the final output. Therefore, it requires to collude with the other computing parties and collect all the computed shared values to achieve its goal. Notably, in this work, our goal is not to design protocols that prevent collusion but to define non-colluding parties for the security guarantee of the protocol so that multiple parties can not combine their views in order to learn extra information.

The second issue is the independence of involved parties in these protocols. If an adversary controls one party, the other parties should behave honestly. We assume that each of the computation and input parties are independent.

Lastly, one or more computation parties may get corrupted. We assume that the corrupted party follows the protocol but may try to learn as much as possible from the messages they receive from other parties and tries to compute the inputs and outputs of honest parties based on all the available information. The corrupted parties are also commonly called honest-but-curious. The number of corrupted computation parties tolerated depends on selected security assumptions.

More specifically, we consider the security of our system under two settings:

- *Honest (non-corrupted) majority* In this setting, the adversary may actively corrupt *t* computation parties, s.t. $t < n/2$. We address this case in a three-party setting where, at most, one party can be corrupted.
- *Dishonest (corrupted) majority* In the second case, the corrupted parties could be the majority, that is the number of corrupted parties could be more than or equal to half of the participants (i.e. $t \ge n/2$). To achieve the highest efficiency, we address this case in the two-party setting where only one of the computation parties can be corrupted.

This paper propose logistic regression training protocols for both of these security settings.

### Secret sharing

Secret sharing is a set of techniques that allows a secret value *x* to be distributed among *n* participants as $x_1, \ldots , x_n$ so that each party $P_i$ receives a random share $x_i$ (*mod* *p*) of the secret over some prime *p*.^1^ In secret sharing-based secure computation schemes, a number of sensitive data holders (input parties) can secretly share their data among other participants. In this paper, we use the n-out-of-n additive secret sharing scheme. In this scheme, an integer *u* is additively shared (meaning the shares sum to *u*) between *n* participants. To do this, each input party picks $n - 1$ randomly generated values and sends them to all other participants. The last party is provided by the secret *u* minus the sum of those randomly generated values. The original value can be reconstructed by computing the summation of all of the shares.

In what follows, we will use $[\![ x ]\!]$ to denote secret shares that reconstruct to *x*. A share $[\![ x ]\!]$ is an n-tuple with each computing party holding precisely one element of the tuple and $[\![ x ]\!]_{i}$ denotes the share held by the $i_{th}$ party.

### Addition and multiplication

Various operations can be performed on secret shared data through the tailored protocols. In accordance with the security models discussed in the preceding “System model” section, in our work, we employ addition and multiplication as the key operations. We closely follow the notation used in [24] to present the protocols to perform these operations.

The addition of two secrets can be performed locally as follows: $[\![ x ]\!] + [\![ y ]\!] = ([\![ x ]\!]_1 + [\![ y ]\!]_1, [\![ x ]\!]_2 + [\![ y ]\!]_2,\ldots , [\![ x ]\!]_n + [\![ y ]\!]_n)$. The multiplication of the additively secret shared values, on the other hand, requires network communication and calls for a different treatment for each security setting we consider.

In the honest majority setting, we use the multiplication protocol proposed by Bogdanov et al. [25] (classical approach) and refined in [24]. In this protocol, the multiplication of two additively secret shared values *x* and *y*, is computed in the three-party setting as follows:

1 $$\begin{aligned} (x_1 +x_2 +x_3)(y_1 +y_2 +y_3)=(x_1y_1 + x_1y_3 + x_3y_1) \\ +(x_2y_2 + x_2y_1 + x_1y_2) +(x_3y_3+x_3y_2+x_2y_3) \\ = \sum _{i=1}^{3}x_{i}y_{i}+x_{i}y_{p(i)}+x_{p(i)}y_{i} \end{aligned}$$

where p(i) indicates the index of the previous computation party. Eq. 1 implies that each computation party requires its adjacent computation party’s input share and if $i = 1$ then the previous party is 3, thus forming a loop. Therefore, each computation party transfers the shares of inputs *x* and *y* it received earlier to the next party to compute the following equation:

$$\begin{aligned} {[}\![ w ]\!]_i = [\![ x ]\!]_i . [\![ y ]\!]_i + [\![ x ]\!]_{p(i)} .[\![ y ]\!]_i +[\![ x ]\!]_i.[\![ y ]\!]_{p(i)} \end{aligned}$$

where $[\![ w ]\!]_i$ is a share of the multiplication result calculated by the computation party *i*. However, as also noted by Bogdanov et al. [25], sending the input share to the nearby computation party may give an advantage to an adversary who may have observed earlier shares. To address this issue, they introduced a re-sharing protocol to construct a new share from an input share for each computation party at the beginning and end of the multiplication operation.

In the dishonest majority setting, we use the Beaver triples technique [26] to perform the multiplication operation. This method requires the presence of a trusted initializer which pre-distributes the shares ($[\![ a ]\!]$, $[\![ b ]\!]$, $[\![ c ]\!]$) of multiplication triple (*a*, *b*, *c*) between the computation parties in such a way that *a* and *b* are randomly generated and $c = a . b$. Once the shares of the input and the triple are received, each computation party computes $[\![ d ]\!]$ = $[\![ x ]\!] - [\![ a ]\!]$ and $[\![ e ]\!]$ = $[\![ y ]\!] - [\![ b ]\!]$ locally and then reveal $[\![ d ]\!]$ and $[\![ e ]\!]$ to other parties. By using these shares, the parties can reconstruct *d* and *e*. Since *a* and *b* are randomly generated, revealing the shares of *d* and *e* does not compromise the security of the protocol. Given these values, each party locally computes:

$$\begin{aligned} {[}\![ w ]\!]_i = [\![ c ]\!]_i +e .[\![ b ]\!]_i + d .[\![ a ]\!]_i +e.d \end{aligned}$$

where $[\![ w ]\!]_i$ is a share of the result of the multiplication calculated by the computation party *i*.

It is important to note that after distributing the shares of the multiplication triple, the trusted initializer will not be involved in the rest of the protocol.

### Matrix inversion

As we will explain in “Methods” section , matrix inversion operation is required in order to implement logistic regression. The secret sharing based protocols discussed in the previous section support only addition and multiplication operations, and the accurate (as opposed to approximate) implementation of matrix inversion with these protocols incurs a significant computational cost. To address this issue, we use the approximation method introduced by Nardi et al. [27]. Nardi’s method converts the matrix inversion problem into an iterative procedure of matrix multiplication and addition. In this method, we look for a matrix *B* that is equal to the inversion of the matrix *X*. The main idea is to define a function *f*(*x*) for which matrix *X* represents its root. More formally:

$$\begin{aligned} f(x) = X^{-1} - B \end{aligned}$$

To find the root of the function *f*, Nardi suggested the use of the Newton–Raphson method [28]. Thus, a stable numerical iterative approximation takes the following form:

2 $$\begin{aligned} B_{s+1} = 2B_s - B_sM_s \quad B_0 = c^{-1}\mathbb {I} \\ M_{s+1} = 2M_s - M_s^2 \quad M_0 = c^{-1}X \end{aligned}$$

where $M_s = B_sA$, $B_0$ and $M_0$ are the initial guesses, $\mathbb {I}$ is an identity matrix, and *c* is a constant. After convergence, $B_{s}$ contains an approximation of matrix *X*’s inversion.

### Logistic regression

Logistic regression is a statistical technique that is commonly used in machine learning tasks. It predicts the probability whether a dependent variable belongs to a particular class. This paper will consider the binary classification, where there are only two possible classes. The logistic model is intended to describe a probability, which is always a number between 0 and 1.

Let $D = \{(X, y)\} = \{(x_1, y_1), (x_2, y_2)$ $,\dots , (x_n, y_n)\}$ be a training dataset of n records, where $x_i$ is the m-dimensional feature vector of each record and the $y_i$ is a vector of labeled binary outcomes. The logistic regression model is given by:

3 $$\begin{aligned} P(y_i=1|x_i;\beta )= {\frac{1}{1-e^{-\beta ^Tx_i}}} \end{aligned}$$

where $\beta = (\beta _1,\ldots , \beta _{m})$ is the m-dimensional regression coefficients vector and $\beta ^T$ is its transpose, $y_i$ is the observation of binary responses and $x_i$ is the feature vector belonging to the record *i*. The purpose of using this method is to obtain the parameter vector $\beta$ that maximizes the log-likelihood function:

4 $$\begin{aligned} l(\beta ) = -\sum ^{n}_{i=1}\log \left( 1+e^{-\beta ^Tx_i}\right) \end{aligned}$$

By determining the parameters $\beta$, the classifier can predict the class label of new feature vectors.

## Methods

### Estimating model coefficients

Since logistic regression cannot be found in a closed form, model estimation is often accomplished by an iterative optimization over the log-likelihood function. As mention in “Matrix inversion” section, Newton–Raphson is a popular numerical iterative method that eventually approaches the optimal values of the model parameters. For each iteration, the coefficient estimates are updated by:

5 $$\begin{aligned} \beta _{new} = \beta _{old} - \mathbb {H}^{-1}(\beta _{old}) \nabla (\beta _{old}) \end{aligned}$$

where $\nabla$ and $\mathbb {H}$ correspond to the gradient and Hessian of the log-likelihood function respectively. They are evaluated with the old estimate of the $\beta$ to determine the current estimate and can be computed as follows:

6 $$\begin{aligned} \nabla (\beta ) = \frac{\partial l(\beta )}{\partial \beta } = \mathbb {X}^\mathbb {T}(y-\pi ) \end{aligned}$$

7 $$\begin{aligned} \mathbb {H}(\beta ) = \frac{\partial ^2 f}{\partial \beta \partial \beta ^\mathbb {T}} = \mathbb {X}^\mathbb {T}\mathbb {W}\mathbb {X} \end{aligned}$$

where $\mathbb {W}$ is a diagonal matrix with elements defined as $a_{i,i} = \pi (1 - \pi )$ and $\pi$ is the vector of probabilities.

### Gradient

As stated in (6), to compute the gradient, we first need to compute the Sigmoid function ($\pi$). The Sigmoid function is a mathematical function that has a characteristic S-shaped curve. This function has the property that maps the entire number line into a small range, such as between 0 and 1.

8 $$\begin{aligned} \pi (z) = \frac{\mathrm {1} }{\mathrm {1} + e^{-z}} = (\mathrm {1} + e^{-z})^{-1} \end{aligned}$$

During the computation of the Sigmoid function, we consider both accurate and approximate cases which are summarized below.

*Accurate Computation* The main challenges of computing the exact value of the Sigmoid function are performing exponentiation and matrix inversion operations. To perform the matrix inversion operation, we use the solutions discussed in “Matrix inversion” section. However, performing exponentiation by the considered secret sharing techniques is quite challenging.

After each computation party receives the other computation parties’ share of $e^{[\![ z_i ]\!]}$, they computes $({{[\![e^{[\![ z_1 ]\!]}]\!]}_1} * {{[\![e^{[\![ z_2 ]\!]}]\!]}_2} * \dots *{{[\![e^{[\![ z_i ]\!]}]\!]}_n}$), which is equal to $([\![{e^{[\![ z_1 ]\!] + {[\![ z_2 ]\!]} + \dots + {[\![ z_i ]\!]}}}]\!])$. Therefore, each computation party has a valid share of $[\![e^{[\![ z ]\!]}]\!]$, and uses the MPC-based addition and matrix inversion operations to compute the exact value of the Sigmoid function.

*Least Squares Approximation* The method to compute the exact value of the Sigmoid function might have scalability issues due to the large number of multiplications. In order to improve the performance, we use the least-squares approximation of the sigmoid function over the interval [-8,8] introduced by Kim et al. [15]. We adapt this approximation method and consider the degree 3, 5, and 7 least-squares polynomials:

$$\begin{aligned} {\left\{ \begin{array}{ll} g_3(x) = 0.5 + 1.20096 . (x/8) - 0.81562 . (x/8)^3 \\ g_5(x) = 0.5 + 1.53048 . (x/8) - 2.3533056 . (x/8)^3 \\ \qquad \qquad + 1.3511295 . (x/8)^5\\ g_7(x) = 0.5 + 1.73496 . (x/8) - 4.19407 . (x/8)^3 \\ \qquad \qquad + 5.43402 . (x/8)^5 - 2.50739 . (x/8)^7 \end{array}\right. } \end{aligned}$$

The degree 3 least-squares approximation requires fewer multiplications, while the degree 7 polynomial has more immeasurable precision.

### Hessian

The Hessian matrix $\mathbb {H}$ denotes the second partial derivatives of the maximum likelihood function $l(\beta )$. In every iteration, the Hessian matrix has to be updated by the newest $\beta$, and its inversion has to be computed. To evaluate the Hessian matrix, we can consider two different methods. First, we can compute the exact value of the Hessian matrix by performing the required MPC-based multiplication. However, the exact evaluation of the Hessian matrix is considerably expensive in computational terms. To solve this issue, we approximate the Hessian matrix with a fixed matrix instead of updating it in every iteration. More specifically, we can replace the fixed Hessian matrix with its approximation $\tilde{\mathbb {H}}$ (Eq. 9) that only needs to be computed and inverted.

9 $$\begin{aligned} \tilde{\mathbb {H}} = \frac{-1}{4}XX^T \end{aligned}$$

Böhning [29] proved that if $\tilde{\mathbb {H}} - \mathbb {H}$ is positive definite and $\tilde{\mathbb {H}} \le H$ then the convergence of this method is guaranteed. Also, because $\tilde{\mathbb {H}}$ does not depend on $\beta$, we can pre-compute the Hessian and its inverse one time and use it in all iterations.

### Privacy-preserving logistic regression training

This work assumes that the result party desires to compute the logistic regression model over collected data by different data owners. Each data owner computes multiple shares (based on the number of computation parties) of its sensitive data and sends them separately to each computation party. Note that each computation party receives an equal number of dependent $X_i$ and independent $y_i$ variables. Each computation party should append the received shares and their corresponding dependent variables in the correct order. Finally, computation parties send their computed shares of logistic regression coefficient to the result party, and the result party, then, simply sum these shares together to compute the final result.

We now present our privacy-preserving logistic regression training algorithms that employ the previously mentioned approaches. These algorithms summarize the crucial steps in the proposed protocols for both honest and dishonest majority security assumptions. In our proposed algorithms, each data owner provides a share of data for the computation parties as input. The only output of the algorithm is the computed model coefficients $\beta$. Notably, we will not employ a convergence check after each iteration to prevent unnecessarily revealing information about the input. $n_{iter}$ specifies the upper bound of the number of iterations needed for convergence.

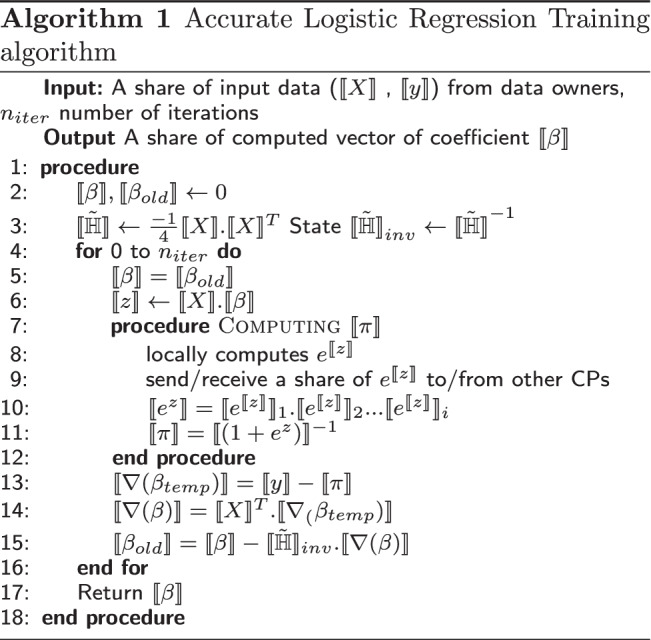



In Algorithm 1, we propose a very accurate privacy-preserving logistic regression model training protocol. In this algorithm, we only employ highly accurate approximations, such as matrix inversion and fixed Hessian, which have a negligible effect on the computation output’s accuracy. Moreover, instead of approximating the Sigmoid function, we use our approach introduced in “Gradient” section to compute the exact value of it (lines 8-12).

The primary purpose of proposing Algorithm 2 is to achieve a highly efficient privacy-preserving logistic regression model training protocol. Various approximation approaches such as fixed Hessian matrix, least-square approximation for the Sigmoid function, and matrix inversion algorithm are employed to obtain our goal. This logistic regression training algorithm demonstrates how the introduced approximation approaches can be combined efficiently to compute the logistic regression coefficient in a privacy-preserving manner.

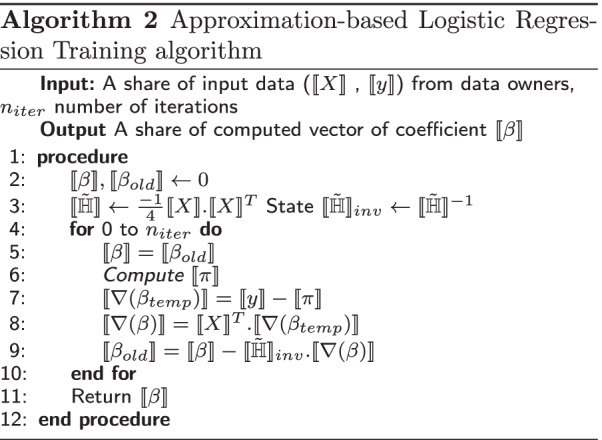



## Results

In this section, we first describe computational efficiency evaluations in terms of CPU time and memory consumption for the proposed algorithms over a real-world dataset and generated synthetic data. We finally describe the accuracy evaluations of these protocols and theoretically discuss the communication cost.

### Implementation details

We implemented our proposed algorithms with both of the introduced security settings in “Implementation details” section using Python programming language. Algorithm 1 is implemented using Beaver triple-based MPC (Accurate BMPC) and classical MPC (Accurate CMPC). Also, the Beaver triple-based version of Algorithm 2 is called BMPC, and the classical-based MPC implementation of this algorithm is called CMPC. Moreover, to have a decent comparison, the ordinary logistic regression (OLR), which does not use MPC, is implemented.

Experiments were performed on an ARM-based M1 processor with 16GB memory, running a macOS operation system. Also, to eliminate the impact of network latency, we simulated the computing nodes on a single computer with multiple threads (each node is considered as a thread). Each experiment was performed at least ten times and its output’s mean was reported. During the validation, we employed both synthetic and real-world datasets.

We report the evaluation results concerning computational efficiency in terms of CPU time and memory consumption and result accuracy. For a fair comparison on the efficiency, we used four real-world data sets: Pima Indians Diabetes Dataset (PIMA) [30], Low Birth Weight Study (LBW) [31], Prostate Cancer Study (PCS) [32], and Umaru Impact Study datasets (UIS)[33]. All datasets have a single binary outcome variable. To satisfy the demand for large-scale studies between multiple research institutions with a large number of records, we also examined our protocols with synthetic data sets of varying sizes. The synthetic data is composed of up to 1 million records spanning up to 3000 features representing most real-world use cases.

### Efficiency

To compare our protocols’ efficiency with an ordinary logistic regression, first, we measured the CPU time of our protocol when the number of features is constant (i.e., 250), and the number of records increases. We, then, calculated the CPU time of the protocols when the number of records is fixed (i.e., 7000), and the number of features increases.

**Fig. 1:**
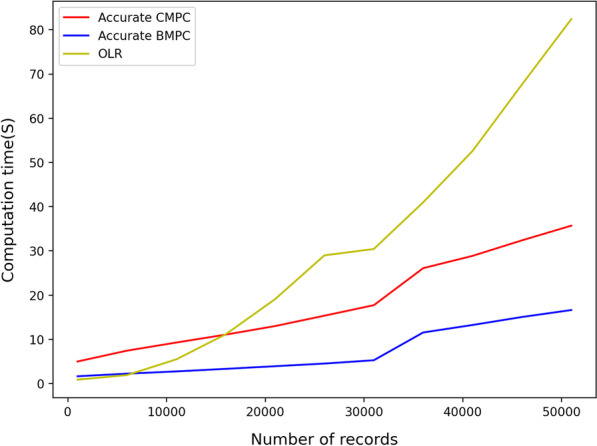
Efficiency comparison for increasing number of records using accurate algorithm 1

**Fig. 2:**
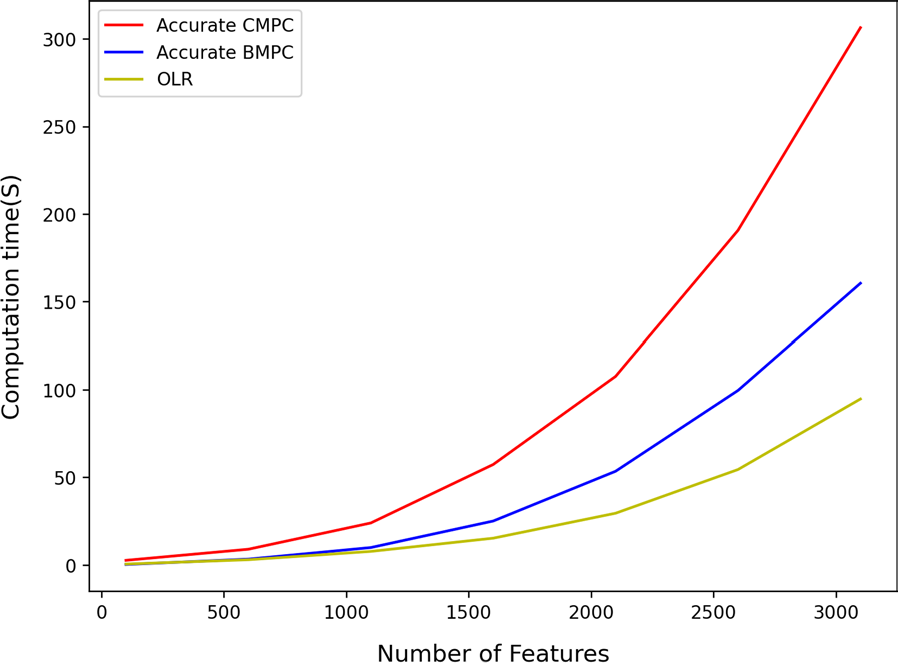
Efficiency comparison for increasing number of features using accurate algorithm 1

The CPU time of the proposed protocols is heavily influenced by the number of records and training set features. Figures 1 and 2 illustrate the CPU time of implemented protocols based on algorithm 1 (Accurate BMPC and Accurate CMPC). As is illustrated in Fig. 1, Accurate BMPC has the best results when the number of records increases. This protocol computes a logistic regression model over a train set of 50000 records and 500 features in less than 15 s which is 20 s faster than Accurate CMPC protocol and 70 s faster than OLR. Nevertheless, increasing the number of features has a higher impact than OLR. As is shown in Fig. 2, using the OLR protocol, a logistic regression model can be trained over a training set with 7000 records and 3000 features in around 90 s. This model, however, takes about 3 minutes and 5 minutes to compute using the Accurate BMPC protocol and Accurate CMPC protocol, respectively.

**Fig. 3:**
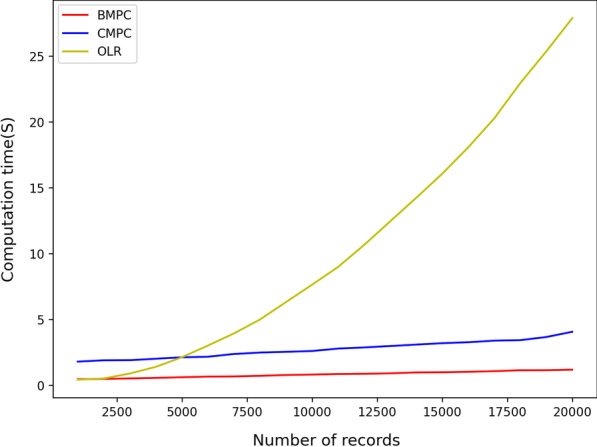
Efficiency comparison for increasing number of records using approximation-based algorithm 2

**Fig. 4:**
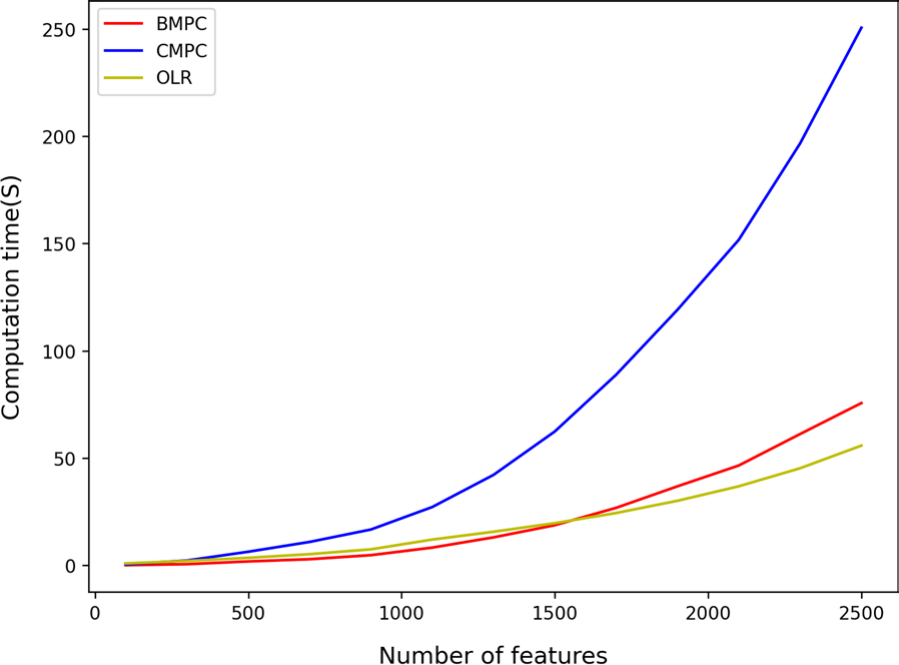
Efficiency comparison for increasing number of features using approximation-based algorithm 2

Figures 3 and 4 illustrate the CPU time of the protocols which are implemented based on algorithm 2 (BMP and CMPC). As shown in Fig. 3, both protocols have a better performance than OLR when the number of records increases and the number of features is fixed. Also, BMPC has a considerably better CPU time in comparison with CMPC and OLR. However, as is shown in Fig. 4, increasing the number of features has slightly different results. Raising the number of features decreases the efficiency of all three protocols. CMPC receives the highest impact from rising the number of features, but BMPC still has an acceptable efficiency level. For example, BMPC can train a model with 7000 records and 2500 features in less than one minute, 7 s higher than OLR, and four times better than CMPC. Therefore, we can conclude that CMPC is not the right choice when we have a dataset with a considerable number of features.

Besides, to measure the efficiency in terms of memory consumption, a python module named “memory_profiler” [34] has been utilized. Table 1 indicates memory consumption for the different introduced protocols and ordinary logistic regression. All implemented protocols in both security settings consume remarkably less memory during the training process. As is described in the Table 1, to train a logistic regression model over a dataset with 50,000 records and 200 features, the OLR protocol requires about 30 GB of memory. However, the BMPC and Accurate BMPC have about 8 GB of memory to train this model. Also, the CMPC and Accurate CMPC protocols have a better consumption rate than BMPC, and less than 5 GB of memory is needed to train such a model.

**Table 1:** Memory consumption comparison of our proposed protocols using generated synthetic datasets

Records number	Feature number	OLR	BMPC	CMPC	Accurate BMPC	Accurate CMPC
10,000	50	3666	1325	688	1316	888
20,000	70	12,559	1717	1016	1665	1325
30,000	90	22,736	2743	1702	2654	2033
40,000	100	29,110	3775	2337	3622	2672
50,000	100	34,394	4575	2878	4406	3220
50,000	200	34,512	8516	5437	8013	5770

### Accuracy

One of the primary subjects that we considered in examining our protocols was accuracy. We measured accuracy based on the estimated model parameters’ precision during the training phase over the Low Birth Weight Study dataset. To do this, we compared the obtained vector of coefficient $\beta$ from our protocols with the ones estimated using the OLR protocol. As Table 2 presents, the Accurate BMPC and Accurate CMPC protocols’ model parameters are almost the same as the model parameters estimated using OLR protocol. Moreover, the model parameters estimated from protocols based on the algorithm 2 (BMPC and CMPC), which employ various approximations, have an acceptable level of accuracy compared to the model parameters estimated using OLR protocol.

**Table 2:** A comparison between model parameters $\beta$ learned using the proposed protocols and ordinary logistic regression protocol over LBW dataset

$\beta$	Ordinary LR	Accurate BMPC	BMPC			Accurate CMPC	CMPC		
			3	5	7		3	5	7
$\beta _1$	0.01574	0.01577	0.01761	0.01580	0.01480	0.01574	0.02214	0.01793	0.01630
$\beta _2$	0.01127	0.01123	0.01171	0.01061	0.01006	0.01127	0.01534	0.01266	0.01166
$\beta _3$	0.78666	0.78152	0.67763	0.62479	0.60392	0.78662	0.95081	0.81191	0.77010
$\beta _4$	$-0.47132$	$-0.46992$	$-0.48975$	$-0.44423$	$-0.42170$	$-0.47131$	$-0.63960$	$-0.52621$	$-0.48340$
$\beta _5$	$-1.32410$	$-1.31870$	$-1.24442$	$-1.13786$	$-1.08974$	$-1.32405$	$-1.68676$	$-1.41595$	$-1.32408$
$\beta _6$	$-0.75584$	$-0.75594$	$-0.86971$	$-0.78142$	$-0.73330$	$-0.75583$	$-1.09894$	$-0.88944$	$-0.80596$
$\beta _7$	$-2.20748$	$-2.20104$	$-2.48191$	$-2.23117$	$-2.09511$	$-2.20743$	$-3.15262$	$-2.56252$	$-2.33208$
$\beta _8$	$-0.96060$	$-0.95756$	$-0.99906$	$-0.90459$	$-0.85667$	$-0.96058$	$-1.30317$	$-1.07358$	$-0.98838$
$\beta _9$	$-0.24569$	$-0.24476$	$-0.21884$	$-0.20160$	$-0.19465$	$-0.24568$	$-0.30509$	$-0.25879$	$-0.24367$

Regarding the introduction of several approximation schemes in BMPC and CMPC protocols, as well as gaining a better understanding of the accuracy of these protocols, we compared the prediction accuracy achieved by these protocols with that obtained from the OLR protocol. To do this, we calculated the percentage (%) of the correct predictions of estimated models produced on four different datasets (25% of training samples were assigned to the test set) in various settings based on the degree of Sigmoid function approximation. All the accuracy measurement results are summarized in Table 3. This table presents the average prediction accuracy percentage when threshold = 0.5 and the AUC (Area Under the Curve), which estimates a binary classifier’s quality. Moreover, based on the information provided in Table 2, varying the Sigmoid function approximation degrees used in BMPC and CMPC protocols do not significantly affect the estimated model’s accuracy over the chosen datasets.

**Table 3:** Accuracy comparison result for real-word datasets with different settings

Dataset	Records num	Feature num	g(x) degree	CMPC		BMPC		OLR	
				Accuracy	AUC	Accuracy	AUC	Accuracy	AUC
			3	71.87%	0.740	71.87%	0.740	71.87%	0.740
PIMA	768	9	5	71.87%	0.741	71.87%	0.740	71.87%	0.740
			7	71.87%	0.741	71.87%	0.741	71.87%	0.741
			No approx	–	–	–	–	71.87%	0.741
			3	81.05%	0.842	81.05%	0.846	80%	0.846
PCS	379	10	5	81.05%	0.845	81.05%	0.847	80%	0.847
			7	81.05%	0.847	81.05%	0.848	81.05%	0.848
			No approx	–	-	–	–	81.05%	0.848
			3	64.58%	0.519	64.58%	0.519	64.58%	0.519
LBW	189	10	5	64.58%	0.519	64.58%	0.519	64.58%	0.519
			7	62.5%	0.519	62.5%	0.517	64.58%	0.519
			No approx	–	–	–	–	62.5%	0.523
			3	73.61%	0.651	73.61%	0.651	73.61%	0.651
UIS	575	9	5	72.91%	0.652	72.91%	0.652	72.91%	0.652
			7	72.91%	0.655	73.61%	0.651	72.91%	0.655
			No approx	–	–	–	–	72.22%	0.656

### Communication cost

In multi-party computation protocols, communication cost depends on multiple elements, such as the number of computations parties, iterations, operations, and security settings. Moreover, the Newton–Raphson method, which is considered as our optimization approach, incurs considerable computation and communication costs on our protocols. In this work, our main focus was to reduce the computation cost by reducing the number of operations using multiple approximations.

Our experiments have been conducted on a single PC and focused on the computation cost. As it is commonly done in the literature [25], we thus provide an indication on the communication cost as well. To do this, first, we compute the communication cost for the main operations we employ, e.g., addition and multiplication, in both honest majority and dishonest majority settings. We then calculate the total communication cost for the entire protocol by aggregating the costs of each individual operation over several iterations.

In MPC-based protocols, addition operation in both security settings does not require any communication between the computation parties. However, multiplication operation requires multiple communication rounds. In the honest majority setting and with three computation parties, Bogdanov [35] explained that each time performing multiplication operation requires exchanging 15 messages between the computation parties. If we consider each message with the size of 32 bits, the communication cost to perform a multiplication operation will be 420 bits. Besides, executing a logistic regression protocol once requires (CMPC) requires ten iterations, and multiplication protocol will be performed in each iteration between 100 and 300 times.^2^ Therefore, to compute the logistic regression model in an honest majority setting with our protocol, at least 420 Kb data will be exchanged.

Communication costs in the dishonest majority setting are notably lower (i.e. due to Beaver triples) than the honest majority setting. The multiplication procedure in this method is split into the offline and online phases. Multiplication triples will be generated and distributed during the offline phase before the computation parties’ inputs are associated. Therefore, there is no communication cost in this phase. During the online phase and in the two-party setting, each computation party sends only two messages to the other party to perform the multiplication operation. If we consider each message with the size of 32 bits, 128-bits data need to be exchanged for one-time multiplication in this setting. Accordingly, the computation time is 3.75 times less than the honest majority setting. For the whole logistic regression protocol with 10 iterations, at least 125 Kb of data will be exchanged.

## Conclusions

There is an increasing interest in applying machine learning algorithms to sensitive data, such as medical data. In this paper, we described novel algorithms for implementing secure and private logistic regression training among distributed parties by using multi-party computation protocols. We evaluated the performance of our algorithms through experiments on real-world and synthetic datasets to show the scalability of our solutions, mainly when they are applied to a dataset with a large number of records and features. Our experiments also showed that our algorithms achieve high accuracy while maintaining a reasonable level of efficiency. As future work, we are planning to extend our approach to support secure and efficient multi-class logistic regression.

## Data Availability

The data used in the present study are publicly and freely available and have been downloaded from public data repositories. All datasets and codes used in this study are freely available at: https://github.com/alirezaghavamipour/pplr_ss

## Abbreviations

LR: Logistic regression
MPC: Multi-party computation
GLORE: Grid binary logistic regression
SMAC-GLORE: Secure Multi-pArty Computation Grid LOgistic REgression
BMPC: Beaver triple-based multi-party computation
CMPC: Classical multi-party computation
OLR: Ordinary logistic regression
PIMA: Pima Indians diabetes dataset
LBW: Low birth weight study
PCS: Prostate cancer study
UIS: Umaru impact study datasets

## Footnotes

1 We drop the modular notation for the sake of conciseness in the rest of the paper.

2 The exact numbers of iterations and multiplications depend on the degree of least-squares Sigmoid approximation and the choice of a good start value for the inversion operations.
